# Management of Cognitive and Autonomic Symptoms in LBD

**DOI:** 10.1002/alz.090527

**Published:** 2025-01-09

**Authors:** Douglas W. Scharre

**Affiliations:** ^1^ Ohio State University, Columbus, OH USA

## Abstract

Support for the management of cognitive and autonomic features of Lewy body dementia (LBD) is offered in detail in this presentation. The US Based Diamond Lewy Cognitive Symptoms management document presents the unique cognitive profile of LBD with general principles for addressing these features along with detailed guidance in the use of cholinesterase inhibitors and Memantine. The US Based Diamond Lewy Autonomic Symptoms management document addresses the management of common autonomic symptoms including urinary dysfunction, male sexual dysfunction, excessive sweating, constipation, sialorrhea, gastroparesis and orthostatic hypotension in LBD.

